# External validity in a multicenter randomized clinical trial of proximal humeral fractures: the DelPhi trial

**DOI:** 10.1007/s00590-021-02982-6

**Published:** 2021-04-20

**Authors:** Elias Tallay, Sondre K. Lindberg, Sindre Lee-Ødegård, Jonas Bjordal, Alexander N. Fraser, Jan Erik Madsen, Tore Fjalestad

**Affiliations:** 1 Division of Surgery, Orthopaedic Department, Oslo University Hospital, Ullevål, Norway; 2 Department of Orthopedic Surgery, Østfold Hospital Trust, Grålum, Norway

## Abstract

**Background:**

Randomized controlled trials (RCT) are regarded as the gold standard for effect evaluation in clinical interventions. However, RCTs may not produce relevant results to all patient groups. We aimed to assess the external validity of a multicenter RCT (DelPhi trial).

**Methods:**

The DelPhi RCT investigated whether elderly patients with displaced proximal humeral fractures (PHFs) receiving reversed total shoulder prosthetic replacement (RTSA) gained better functional outcomes compared to open reduction and internal fixation (ORIF) using an proximal humerus locking plate (PHILOS). Eligible patients were between 65 and 85 years old with severely displaced 11-B2 or 11-C2 fractures (AO/OTA-classification, 2007). We compared baseline and follow-up data of patients for two of the seven hospitals that were included in the DelPhi trial (*n* = 54) with non-included patients (*n* = 69). Comparisons were made based on reviewing medical records regarding demographic, health and fracture parameters.

**Results:**

Forty-four percent of the eligible patients were included in the DelPhi trial. Comparing included and non-included patients indicated higher incidences of serious heart disease (*P* = 0.044) and a tendency toward higher tobacco intake (*P* = 0.067) in non-included patients. Furthermore, non-included patients were older (*P* = 0.040) and had higher ASA classification (*P* < 0.001) and were in more need for resident aid (in-home assistance) (*P* = 0.022) than included patients. The cause of PHF was more frequently related to fall indoors in non-included vs. included patients (*P* = 0.018) and non-included patients were more prone to other concomitant fractures (*P* = 0.004). Having concomitant fractures was associated with osteoporosis (*P* = 0.014). We observed no significant differences in rates of complications or deaths between included and non-included patients within 3 months after treatment. In descending order, non-included patients were treated conservatively, with PHILOS, RTSA, anatomic hemi-prothesis or an alternative type of ORIF. RTSA was the preferred treatment choice for C2-type fractures (*P* < 0.001).

**Conclusions:**

Results from the DelPhi RCT may not directly apply to older PHFs patients with lower health status or concomitant fractures.

**Level of evidence:**

Level 4.

## Introduction

With increased life span of the world population, the predicted incidences of proximal humeral fractures (PHFs) will rise. Fractures represent a considerable burden for the patients in terms of pain, loss of function and mortality [1]. PHFs make self-care and independent living difficult and are associated with reduced quality of life [2–6].

Several studies have evaluated treatment effects for PHFs, but most of them represent either low level of evidence [7], leaving several important issues unresolved [8, 9], or lack proper blinding, follow-up duration, and standardized validated outcome measures [8, 9]. The DelPhi trial [10] was designed and conducted to overcome these issues, and intended to represent the highest level of evidence on PHF treatment so far. Briefly, DelPhi is a multicenter semi-blinded randomized controlled trial comparing two operative methods for displaced PHFs, reversed total shoulder arthroplasty (RTSA) vs. open reduction internal fixation (ORIF) [10, 11].

In clinical decision making, orthopedic surgeons need to evaluate results from relevant studies, such as the DelPhi trial. The relevance of the results from clinical studies depends on applicability for a defined set of patients in a specific clinical setting, known as generalization or external validity [12]. The external validity of a study depends on the patient sample being representative of the group of patients in question. Most often, only a small portion of eligible patients are included in RCTs [12]. This may lead to sampling bias, indicating that some patients were more likely to be included than others, resulting in a non-random study sample.

Studies from other areas within medicine, such as osteoporosis [13], have shown poorer health status, lower socioeconomic status, increased cognitive impairment, higher mortality rates and higher cancer rates in non-included vs. included patients [9, 12, 14, 15]. Thus, the actual study sample may differ from the intended population. Evaluation of external validity is therefore of importance because effects of a treatment may vary significantly depending on such differences [12].

In the present study, we investigated potential differences in baseline characteristics, such as health status, incidences of complications and mortality, during follow-up of the first 3 months between included and non-included elderly patients with displaced PHFs in the DelPhi trial [10].

## Materials and methods

Demographic and fracture variables were obtained from the hospital medical records. In the DelPhi trial [11], 124 patients were randomized (46%) of a total of 270 patients assessed for eligibility (146 excluded: 103 did not meet inclusion criteria, 31 declined to participate and 12 due to other reasons). In this study, we compared included patients (*n* = 54) vs. non-included patients (*n* = 69) from two of the seven collaborating hospitals in the DelPhi study: Oslo University Hospital (OUS) and Østfold Hospital Kalnes (ØHK). We included baseline data and 3-month follow-up data for all patients.

The study was approved by the Regional Committee of Research, Health Region Southeast, Oslo, Norway, on November 6, 2012 (Reference 2012/1606). Patients in both groups gave their written informed consent. The DelPhi trial [11] was first issued in November 20, 2012, and registered at ClinicalTrials.gov with identifier: NCT01737060.

## Inclusion and exclusion criteria

The DelPhi trial [11] included Norwegian-speaking patients between 65 and 85 years with displaced three- or four-part proximal humeral fractures (11-B2 or 11-C2) according to AO/OTA-classification (2007 revision) [16] based on both radiographs and CT scans.

Exclusion criteria were previous history of injury or illness of any shoulder, injuries to other parts of the humerus or contra-lateral upper extremity, alcohol or drug abuse, dementia, neurological diseases, glenoid fracture or deformity, head-split fractures, fracture dislocations and high-energy trauma. Non-Norwegian-speaking patients, or patients that for any reason were deemed as non-compliant to rehabilitation, were not included in the DelPhi study.

In the current study, we compared patients that were included in the DelPhi study with *non-included* patients from two participating hospitals (OUS and ØHK). The *non-included* patients were within the same fracture group and age group, but not eligible according to DelPhi exclusion criteria.

## Demographic and fracture variables

The included and non-included patients were compared with regard to age, sex, previous fractures, ASA score (https://www.asahq.org/resources/clinical-information/asa-physical-status-classification-system) and residential status. Previous fractures were registered, and included radius, ulna, humerus, spine, femur and tibia. The need of resident aid was defined as either none, receiving public service at home or living in an institution. Pre-morbidity included mild heart disease (hypertension or mild angina pectoris), more severe heart disease (compensated failure or valve disease), use of prednisolone (> = 10 mg daily), chronic obstructive pulmonary disease (COPD), rheumatoid arthritis, diabetes, osteoporosis diagnosed with DEXA scan or receiving specific medication or low energy fracture with former osteoporotic fracture, smoking (> 10/day) or other relevant conditions such as Alzheimer´s disease and cancer. Concomitant injuries would include soft tissue damage (muscle, ligament, tendon or any type of organ damage) and other fractures. Days from injury to surgery were counted.

## Adverse events 3 months of follow-up

Included and non-included patients were also compared with regard to complications following treatment; for patients treated with ORIF failed fixation, increasing fracture displacement or screw penetration into the gleno-humeral joint. For those treated with arthroplasty dislocation, sign of implant loosening (stem or glenosphere) or peri-prosthetic fracture. For all patients, infections and cardio-pulmonary incidents were noted. Number and time of deaths were obtained from the hospitals’ electronic patient records, connected to the national Norwegian population registry. Norwegian population mortality data are considered complete (http://ssb.no/en/dode/).

## Evaluation of treatment options for B2 and C2 fractures

The treatment options available to treat displaced B2 or C2 fractures are mainly non-operative or operative with a variety of implants; ORIF with locking plate (e.g., PHILOS) or intramedullary nails, hemi-arthroplasty or reversed total shoulder arthroplasty [8]. We evaluated the preferred treatment choice made by surgeons by analyzing data from non-included patients.

## Injury mechanisms

Recorded categories were: fall in- or outdoors, injuries related to sport activities or during any type of transport.

## Statistics

Data were obtained from two out of seven recruiting hospitals; OUS and ØHK, and analyzed using generalized linear mixed regression in R v3.3.1 [17]. Potential confounding effects because of different recruiting hospitals (OUS or ØHK) were assessed by comparing the regression model correcting for different hospitals to a corresponding regression model with no correction for different hospitals. The two regression models were then compared using analysis of variance (ANOVA). Significance was defined at α = 0.05 and two-way tests were performed.

## Results

Subjects demographics are presented in Table 1.

**Table 1:** Demographics

	Included (*n *= 54)	Non-included (*n* = 69)	OR [95%CI]	*P-*group	*P-*hospital
Hospital (ØHK/OUS)	26/28	33/36			
AO/OTA group (B2/C2)	20/34	34/35	1.65 [0.80, 3.41]	0.176	1.000
Age (median [IQR])	73 [11]	77 [10]		0.052	1.000
Sex (male/female)	7/47	14/55	0.59 [0.22,1.57]	0.287	1.000
Previous fractures (yes/no)	32/22	38/31	1.19 [0.58, 2.44]	0.642	1.000
Fracture side (right/left)	29/25	38/31	0.95 [0.46, 1.93]	0.880	1.000
Dominant arm (right/left)	2/19	1/5	1.90 [2.48, 21.6]	< 0.001***	< 0.001***
ASA classification (median [IQR])	2 [0]	3 [1]		< 0.001***	1.000
Days to surgery (mean (SD))	4.6 (2.0)	5.6(4.3)		< 0.001***	1.000
*Pre-morbidities*					
Mild heart disease (yes/no)	31/23	22/47	0.32 [0.15, 0.70]	0.004*	0.022
Serious heart disease (yes/no)	3/51	19/50	6.46 [1.80, 23.2]	0.004*	1.000
Prednisolone (yes/no)	4/50	0/69	Inf	0.925	1.000
COPD (yes/no)	5/49	5/64	0.77 [0.21, 2.79]	0.686	1.000
Rheumatoid arthritis (yes/no)	0/54	4/65	Inf	1.000	1.000
Diabetes wo/insulin (yes/no)	2/52	4/65	1.60 [0.28, 9.08]	0.596	1.000
Diabetes w/insulin (yes/no)	3/51	5/64	1.33 [0.30, 5.82]	0.707	1.000
Osteoporosis (yes/no)	13/41	14/55	0.79 [0.33, 1.92]	0.608	0.033*
Smoking (yes/no)	5/48	12/57	2.04 [0.67, 6.23]	0.212	0.277
Other (yes/no)	47/7	61/8	1.14 [0.38, 3.36]	0.818	1.000
*Resident aid*					
Institution (yes/no)	0/54	4/65	Inf	0.956	0.252
Home aid (yes/no)	2/52	13/56	6.04 [1.30, 28.0]	0.022*	1.000
*Causes of injury*					
Fall indoors (yes/no)	20/34	38/31	0.42 [0.20, 0.86]	0.018*	1.000
Fall outdoors (yes/no)	25/29	14/55	1.39 [0.64, 3.01]	0.408	1.000
Sports (yes/no)	2/52	4/65	1.60 [0.28, 9.08]	0.596	1.000
Multi trauma (yes/no)	0/54	4/65	Inf	0.998	0.370
Unknown (yes/no)	6/48	9/60	1.20[0.40, 3.61]	0.745	1.000
*Concomitant injuries*					
Fractures (yes/no)^1^	0/54	12/57	Inf	0.004**	0.975
Soft tissue (yes/no)	48/6	61/8	0.95 [0.31, 2.94]	0.934	0.855
None (yes/no)	48/6	46/23	0.25 [0.09, 0.67]	0.006**	1.000

*P < 0.05, **P < 0.01 and ***P < 0.001 for non-included vs. included patients. *ØHK* Østfold hospital Kalnes. *OUS* Oslo University Hospital. *OR* odds ratio. CI; 95% confidence interval. *IQR* inter-quartile range. SD; standard deviation. *COPD* chronic obstructive pulmonary disease. *ASA* American Society of Anesthesiologists. Inf; A cell sum was zero, the *P* value should be interpreted with care. *IQR* Inter-quartile range. *P*-group; difference between included and non-included patients. *P*-hospital; *p* value for the difference between patients recruited at OUS or ØHK. ^1^Other than upper extremities

Fifty-four of the 123 eligible patients were included (44%), based on data available for this sub-study from the DelPhi trial (Table 1). The ØHK and OUS hospitals recruited similar numbers of patients (Table 1), and had similar rates of included patients (44% for both hospitals).

There were no significant differences in the distribution of sex, fracture types (AO/OTA group), fracture side, dominant arm and incidence of previous fractures between included and non-included patients (Table 1). Non-included patients tended to have higher age than included patients (*P* = 0.052), and had higher ASA classification, were in higher need for home aid services, as compared to included patients (Table 1). Non-included patients had higher incidences of both mild and serious heart diseases, and tended to smoke more, as compared with included patients (Table 1). The time from injury to surgery was on average 1 day longer for non-included vs. included patients (Table 1).

The cause of PHF was more frequently related to an indoor fall in non-included vs. included patients (Table 1). Non-included patients were also more prone to experience concomitant fractures, as opposed to included patients (Table 1). Experiencing concomitant fractures was highly correlated to having osteoporosis (*P* = 0.014), rheumatoid arthritis (*P* = 0.027) and COPD (*P* = 0.039), using multiple linear regression.

## Adverse events

In the first 3 months of follow-up, the following adverse events were reported for the included patients (*n* = 54) and non-included patients (*n* = 69) that were assessed in this study: There were two failures of the osteosynthesis in the included group and one in the non-included group; one case of screw penetration in the included group and 4 cases in the non-included group; two cases of infection in the included group and three cases in the non-included group; one cardiopulmonary event in the non-included group; no deaths in either group.

## Treatment preferences for non-included patients

Patients included in the DelPhi study were randomized and treated operatively with either RTSA or PHILOS. Out of the 69 non-included patients in the current study, 29 patients were treated conservatively and 40 patients were treated operatively (Fig. 2). In descending order, the most frequent treatment for non-included patients were: conservative (*n* = 29), PHILOS (*n* = 26), RTSA (*n* = 10), anatomic hemi-arthroplasty (*n* = 3) and other ORIF (*n* = 1). Comparing treatment choices for B2 vs. C2-type fractures among non-included patients indicated a preference for using RTSA for C2-type fractures (OR[95%CI]: 0.20[0.08,0.46], *P* = 0.0002) (Fig. 2).

## Potential effects of different recruiting hospitals

**Fig. 1:**
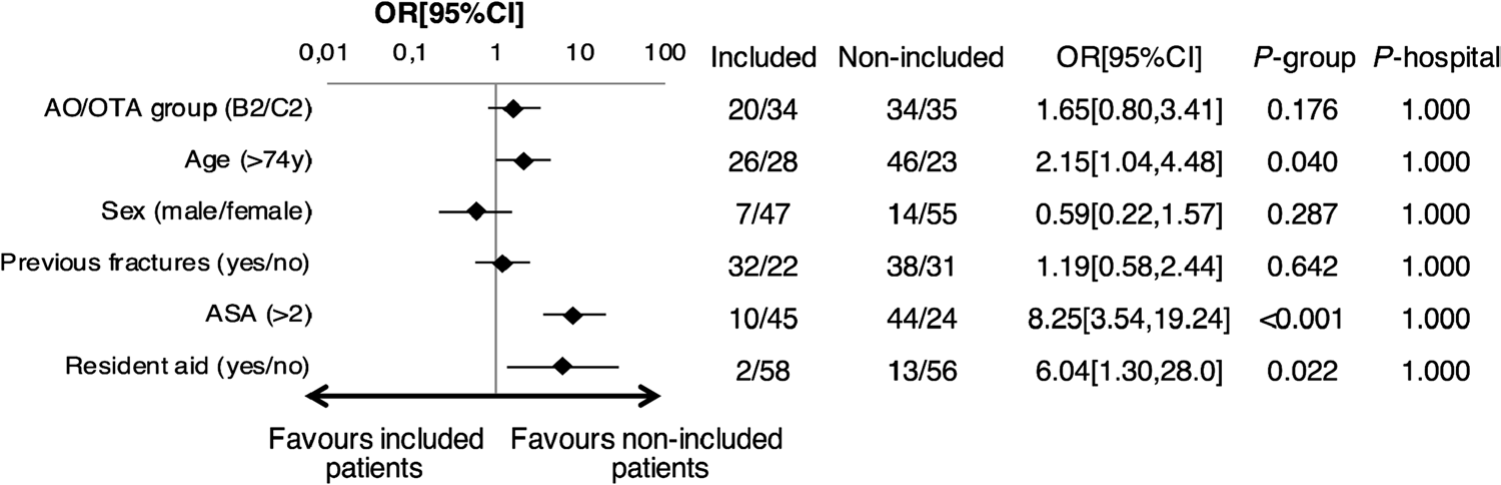
Subject characteristics. Comparison between non-included vs. included patients at baseline. OR; odds ratio. CI; confidence interval. *P*-group; difference between included and non-included patients. *P*-hospital; difference between OUS and ØHK. OA/OTA; AO-Müller/Orthopaedic Trauma Association. ASA; American Society of Anesthesiologists

**Fig. 2:**
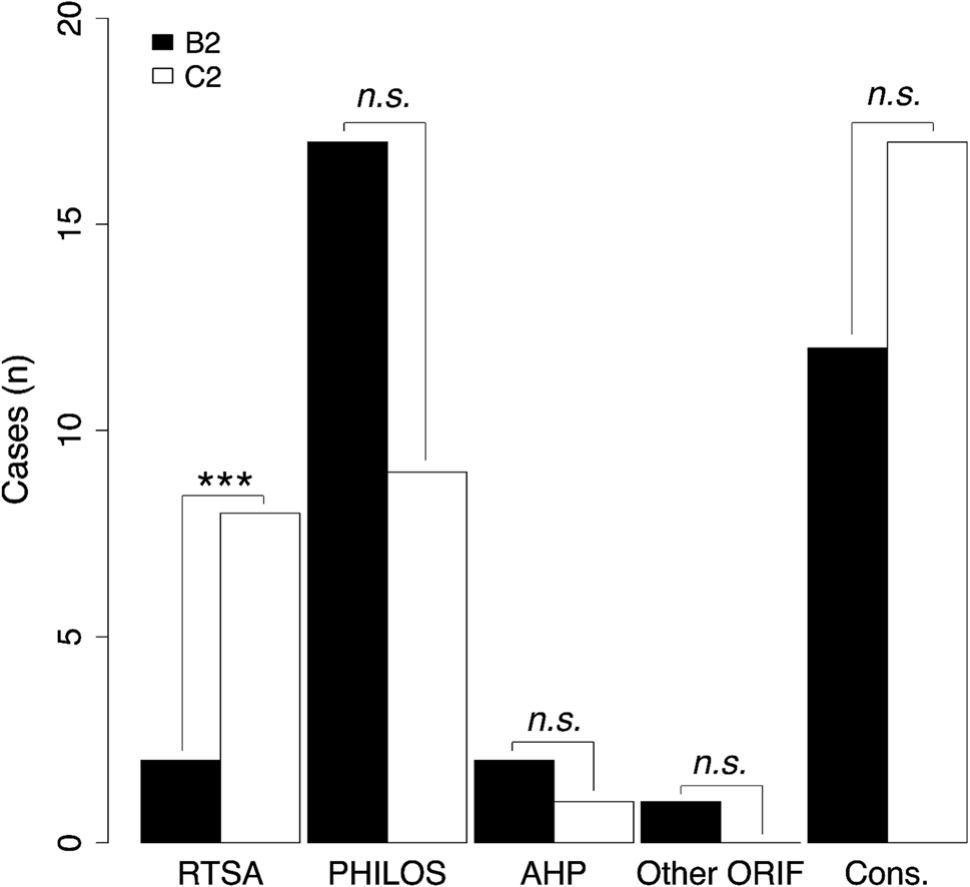
Treatment choices within non-included patients. Comparison of numbers of patients with PHFs receiving different treatments depending on B2- or C2-type fractures. ***P < 0.001. n.s. = not significant. RTSA; reversed total shoulder prosthetic replacement. PHILOS; proximal humerus internal locking system. AHP; anatomic hemi-prothesis. ORIF; open reduction and internal fixation. Cons.; conservative

Because precise data were available from only two of the seven recruiting hospitals, we analyzed possible systematic differences between different recruiting hospitals (Table 1, Figs. 1, 2). At OUS, more patients were registered as right hand dominant and were more likely to have osteoporosis, as compared to ØHK (Table 1). In non-included patients, treatment with RTSA was more commonly chosen at OUS than at ØHK (OR [95%CI]: 1.48 [0.47,2.56], *P* = 0.0052), whereas treatment with an alternative type of ORIF was less commonly chosen at OUS than at ØHK (OR[ 95%CI]: −.44 [ −2.50, −0.42], *P* = 0.007).

## Discussion

This study aimed to evaluate external validity of the DelPhi trial [11] by assessing potential differences between included and non-included patients. Our main findings were that non-included patients were older and displayed higher incidences of heart disease, need of resident aid services and concomitant fractures, as compared to included patients.

Lack of external validity may be a major reason for not implementing demonstrated beneficial treatments into clinical practice [12]. Reduced external validity often occurs as a consequence of strict inclusion criteria in RCTs, increasing the number of non-included subjects. This high rate of non-included patients may be problematic if, e.g., subject characteristics, diseases or disease risk factors differ between the included and non-included populations [18]. This is known as sampling bias; the study population is non-random and results based on this population cannot be generalized. Sampling bias is problematic, e.g., because treatment effects may vary depending on differences in baseline parameters of the study groups [12]. In the DelPhi trial, only 44% of the eligible patients met the criteria for inclusion. Although several baseline characteristics were similar between included and non-included patients, some parameters indicated lower health status in non-included patients, such as higher ASA score, higher prevalence of heart disease and higher need for resident aids. These results from DelPhi are in line with previous reports on non-included patients from other disease areas, such as osteoporosis [13], which also reported lower health status in non-included patients [9, 12, 14, 15]. Some of these studies also reported lower socioeconomic status, increased cognitive impairment, higher mortality rates and higher cancer rates in non-included patients [9, 12, 14, 15]. Despite some studies not reporting such differences [19, 20], it is reasonable to claim that, in general, non-included patients have lower health status compared to included patients in clinical RCTs.

Differences in health status are clinically relevant and may reflect underlying pathology, different stages in the natural history of disease, comorbidities and absolute risk of poor outcome [21]. Furthermore, reduced health status may predict lower treatment compliance [22]. Thus, we assessed if non-included patients experienced more complications during injury or within 3 months after treatment. Our data indicated that non-included patients with PHFs also suffered more concomitant fractures than the included patients, in line with a previous study on osteoporosis? Based on multiple regression analysis of our data, one reason for the higher incidence of concomitant fractures was having osteoporosis. Also, however, an inclusion criterion in DelPhi was absence of injury to other parts of the humerus or contra-lateral upper extremity. Regarding rates of post-treatment complications, we did not observe differences between included and non-included patients in the first 3 months. Taken together, lower health status was associated with concomitant fractures and osteoporosis, and the treatment choice for these types of patients cannot be elaborated upon by results from DelPhi.

To assist in guiding the treatment choice for patients with reduced health status and/or concomitant fractures, we compared treatment choices within non-included patients only. Although conservative treatment was the most frequent separate treatment modality (*n* = 29) for the non-included patients, the total of other operative treatments (*n* = 40) represent the majority of cases, and thus this data does not support prior reporting that non-operative treatment is preferred in the elderly [23, 24].

Among the non-included patients treated with RTSA, there were significantly more type C2 fractures compared with B2 fractures.

The main limitation of this study is that data were available from only two of the seven hospitals involved in the DelPhi trial [11]. However, identical procedures were implemented at all collaborating hospitals in DelPhi, and all hospitals had frequent collaborating meetings. Furthermore, the inclusion rates were identical at both hospitals (44% for both OUS and ØHK). In addition, we assessed potential systematic bias between the two hospitals, revealing some differences in the registration of dominant hand, incidence of osteoporosis and numbers of performed RTSA and ORIF interventions. Although these differences were minor, an improvement to our study would have been the inclusion of data on all *non-included* patients. Furthermore, precise data for the *non-included* patients were limited to the first 3 months, therefore we cannot make conclusions about events later on.

Results from the DelPhi RCT may not be directly applicable to patients with high age, high ASA classification, heart disease, osteoporosis or concomitant fractures. In these patient groups, here represented by the non-included patients, 1/3 of patients with B2 fractures and 1/2 of the patients with C2 fractures and were treated conservatively. When treated operatively, the B2 fractures were most frequently treated with PHILOS, while C2 fractures were treated equally frequent with RTSA as PHILOS.

## Data Availability

By request to the authors.
